# Perioperative fluid and volume management: physiological basis, tools and strategies

**DOI:** 10.1186/2110-5820-1-2

**Published:** 2011-03-21

**Authors:** Mike S Strunden, Kai Heckel, Alwin E Goetz, Daniel A Reuter

**Affiliations:** 1 Center of Anesthesiology and Intensive Care Medicine, Department of Anesthesiology, Hamburg-Eppendorf University Medical Center Martinistraße 52, 20246 Hamburg, Germany; 2 Cardiovascular Research Center, Hamburg-Eppendorf University Medical Center Martinistraße 52, 20246 Hamburg, Germany

## Abstract

Fluid and volume therapy is an important cornerstone of treating critically ill patients in the intensive care unit and in the operating room. New findings concerning the vascular barrier, its physiological functions, and its role regarding vascular leakage have lead to a new view of fluid and volume administration. Avoiding hypervolemia, as well as hypovolemia, plays a pivotal role when treating patients both perioperatively and in the intensive care unit. The various studies comparing restrictive vs. liberal fluid and volume management are not directly comparable, do not differ (in most instances) between colloid and crystalloid administration, and mostly do not refer to the vascular barrier's physiologic basis. In addition, very few studies have analyzed the use of advanced hemodynamic monitoring for volume management.

This article summarizes the current literature on the relevant physiology of the endothelial surface layer, discusses fluid shifting, reviews available research on fluid management strategies and the commonly used fluids, and identifies suitable variables for hemodynamic monitoring and their goal-directed use.

## Introduction

There is increasing evidence that fluid management influences patient's outcome as well in critical illness, as during and after major surgery. Hence, the numerous different aspects contributing to fluid management have been in the focus of both basic and clinical research during the past years. Basically three questions are intrinsically tied to fluid administration perioperatively and in critically ill patients: 1) What happens to intravascular fluid in health and disease? 2) How do different intravenous fluids behave after application? 3) What are the goals for volume administration and how can they be assessed and reached? Current basic research brought fascinating insights of the function of the endothelial vascular barrier and, in particular, regarding functional changes that lead to vascular leakage. Experimental and clinical trials investigating the effects of both crystalloid and colloid solutions--and their natural and artificial representatives--have shown quite conflicting results. The same accounts for the mainly clinical studies that primarily focussed on clinical goals to guide perioperative volume therapy. However, all of those three aspects cannot be separated from each other when defining rational strategies for fluid management. Thus, this review article summarizes the knowledge of the function and dysfunction of the endothelial vascular barrier, on the effect of different intravenous fluids and on the opportunities of hemodynamic monitoring to enable drawing conclusions for rational concepts of perioperative fluid and volume management.

## The underlying aspects

### The physiologic basis: why does fluid stay within the vasculature?

Two thirds of human body fluid is located in the intracellular compartment. The remaining extracellular space is divided into blood plasma and interstitial space. Both compartments communicate across the vascular barrier to enable exchange of electrolytes and nutriments as the basis for cell metabolism. The positive intravascular pressure continuously forces blood toward the interstitial space. Under physiologic conditions, large molecules, such as proteins and colloids, cannot cross the barrier in relevant amounts, which is a necessity for the regular function of circulation. Otherwise, the intravascular hydrostatic pressure would lead to uncontrollable loss of fluid toward the interstitial space and disseminated tissue edema [1]. In 1896, Ernest Starling suggested an interstitial colloid osmotic pressure far below the intravascular pressure. The concentration gradient across the vascular barrier generates a flow, which is directed into the vasculature and opposes the hydrostatic pressure resulting in an only low filtration per unit of time. According to the Starling principle, only the endothelial cell line is responsible for the vascular barrier function [1]. In a rat microvessel model, it has been shown that the interstitial colloid osmotic pressure was nearly 70% to intravascular osmotic pressure without causing interstitial edema, which is in contrast to the Starling's concept, suggesting an only minor role for the interstitial protein concentration [2]. The endothelial glycocalyx plays a pivotal role in this context. Every healthy vascular endothelium is coated by transmembrane syndecans and membrane-bound glypicans containing heparan sulfate and chondroitin sulfate side chains, which together constitute the endothelial glycocalyx [3,4]. Bound plasma proteins, solubilized glycosaminoglycans, and hyaluronan are loading the glycocalyx to the endothelial surface layer (ESL), which is subject of a periodic constitution and degradation. Under physiologic conditions, the ESL has a thickness of approximately 1 μm and binds approximately 800 ml of blood plasma, so plasma volume can be divided into a circulating and noncirculating part [4,5]. Accordingly, the glycocalyx seems to act as a molecular filter, retaining proteins and increasing the oncotic pressure within the endothelial surface layer. A small space between the anatomical vessel wall and the ESL remains nearly protein-free [2]. Thus, fluid loss across the vascular barrier is limited by an oncotic pressure gradient within the ESL [6]! Starlings' classic principle was therefore modified to the "double-barrier-concept" in which not only the endothelial cell line but primarily the endothelial surface layer constitutes the vascular barrier [6].

### Vascular barrier dysfunction: reasons and consequences

The ESL constitutes the first contact surface between blood and tissue and is involved in many processes beside vascular barrier function, such as inflammation and the coagulation system. A number of studies identified various agents and pathologic states impairing the glycocalyx scaffolding and ESL thickness. In a genuine pig heart model, Chappell et al. demonstrated a 30-fold increased shedding of heparan sulphate after postischemic reperfusion [7]. These data were approved by a clinical investigation, which showed increased plasma levels of syndecan-1 and heparan sulphate in patients with global or regional ischemia who underwent major vascular surgery [8]. Beside ischemia/reperfusion-injury, several circulating mediators are known to initiate glycocalyx degradation. Tumor necrosis factor-(α), cytokines, proteases, and heparanase from activated mast cells are well-described actors in systemic inflammatory response syndrome leading to reduction of the ESL thickness, which triggers increased leucocyte adhesion and transendothelial permeability [7,9,10]. Interestingly, hypervolemia also may cause glycocalyx impairment mediated by liberation of atrial natriuretic peptide [11]. Hypervolemia resulting from inadequately high fluid administration therefore may cause iatrogenic glycocalyx damage. As shown in basic research, the dramatic consequence of a rudimentary glycocalyx, which loses much of its ability to act as a second barrier, is strongly increased transendothelial permeability and following formation of interstitial edema [7,11]. The relevance of these experimental data were impressively underlined by Nelson et al., who found increased plasma levels of glycosaminoglycans and syndecan-1 in septic patients, whereas median glycosaminoglycan levels were higher in patients who did not survive [12].

### Fluid balance: where does fluid get lost?

Urine production and insensible perspiration are physiologically replaced by free water absorbed from the gastrointestinal system and primarily affect the extravascular space, if they are not pathologically increased. Because the physiologic replacement is limited in fasted patients, it has to be compensated artificially by infusing crystalloids. The composition of the used infusion should be similar to the physiologic conditions to avoid acid-base disorders, which mostly accounts for balanced crystalloid infusions. During surgery, trauma or septic shock additional fluid loss (blood loss, vascular leakage) affects mainly the intravascular compartment [13,14]. Consequently, the first type of fluid loss is attenuated by redistribution between intracellular, interstitial, and intravascular space slowly and causes dehydration, whereas the second type of loss leads to acute hypovolemia. Preoperative hypovolemia after an overnight fasting period, as described in anesthesia text books [15,16], cannot be explained by the considerations above and does not occur regularly in all patients [17]. Fluid reloading is unjustified, at least in cardiovascular healthy patients before low-invasive surgery [17]. Mediated by increased liberation of atrial natriuretic peptide, undifferentiated fluid loading can cause glycocalyx degradation, increase vascular permeability, promote tissue edema formation and therefore may constitute a starting point of the vicious circle of vascular leakage and organ failure [11,18]. Fluid loss from insensible perspiration also is obviously overestimated in many patients, although loss of only 1 ml/kg per hour occurs even when the abdominal cave is opened [19]. In theory, it should be adequate to substitute only the losses described earlier to maintain a normal blood volume in the critically ill patient. Based on the assumption that a generous fluid administration could prevent hypotension and postoperative renal failure, frequently much greater amounts are infused perioperatively [20], although there is no evidence that the incidence of renal failure is decreased by a liberal infusion regimen during surgery [21]. Furthermore, prophylactic crystalloid infusion does not influence the occurrence of hypotension caused by vessel dilatation [22]. Nevertheless, patients require much more intravenous fluids than suggested by physiologic considerations. Shown by blood volume measurements, major surgery causes a deficit of 3-6 liters in the perioperative fluid balance [23,24]. The peak even persists up to 72 hours after trauma or surgery [25]. The common explanation for this phenomenon is a fluid shift into the so-called third space. This third space can be divided into an "anatomic" and a "nonanatomic" part. Physiologic fluid shifting from the vessel toward the interstitial space across an intact vascular barrier contains only small amounts of proteins. It does not cause interstitial edema as long as it can be quantitatively managed by the lymphatic system. Losses into the "anatomic" third space are based on this mechanism but in a pathologic quantity [13,14], which transgresses the capacity of the lymphatic system. The nonanatomic third space, in contrast, is believed to be a compartment separated from the interstitial space [13,14]. Losses toward this compartment are assumed to be trapped and lost for extracellular exchange. Cited examples for nonanatomic third space losses are fluid accumulation in traumatized tissue, bowel, or peritoneal cavity [15,16], but despite intensive research, such a space has never been identified! Fluid is shifted from the intravascular to the interstitial space! This fluid shift can be classified into two types [13]:

Type 1, occurring always and even if the vascular barrier is intact, represents the physiologic, almost protein-free shift out of the vasculature. Occasionally it emerges at pathologic amounts.

Type 2, the pathologic shift is caused by dysfunction of the vascular barrier. In contrast to type 1, fluid crossing the barrier contains proteins close to plasma concentration [13]. This shift has basically three reasons. First, surgical manipulation increases capillary protein permeability excessively [26]. Interstitial fluid raised approximately 10% during realization of an enteral anastomosis in a rabbit without any fluids being infused [27]. Concomitant administration of 5 ml/kg of crystalloid infusion even doubled this edema. Second, reperfusion injury and inflammatory mediators compromise the vascular barrier [7-10]. Third, iatrogenic hypervolemia can lead to glycocalyx degradation and cause an extensive shift of fluid and proteins toward the tissue [23,24]. The pathologic shift affects all intravenous fluids. Opposed to the common believe that, in contrast to crystalloids, colloids would stay within the vasculature, Rehm et al. described a volume-effect >90% only when a tetrastarch solution was infused titrated to the actual intravascular volume loss. Administered as a bolus in a normovolemic patient, two thirds of the infused volume left the vasculature immediately [23,24]. Volume resuscitation with colloids obviously requires careful titration to current losses to avoid a remarkable protein shift toward the interstitial space [14]. Based on the double-barrier concept, hypoproteinemia even intensifies a vascular barrier dysfunction and promotes tissue edema formation. Perioperative fluid shifting is reflected in clinical data published two decades ago. Lowell et al. showed a weight gain of more than 10% in >40% of patients admitted to the intensive care unit after major surgery. This increase of body weight, representing interstitial edema, correlated strongly with mortality [28].

### Dehydration or hypovolemia?

Dehydration, affecting primarily the extravascular compartment, and acute hypovolemia are two different diagnoses and deserve different therapeutic considerations. Urine production and insensible perspiration cause a loss of colloid-free fluid, which, due to redistribution between intravascular and extravascular space, does normally not impair the intravascular compartment directly. Thus, the resulting dehydration has to be treated by refilling the extravascular space and replacing further losses by crystalloid administration [13]. In contrast, acute hypovolemia at first affects the intravascular compartment. Because crystalloids distribute freely between interstitial and intravascular space, they are not suitable for volume resuscitation in acute hypovolemia. Lost colloids and proteins cause a decreased intravascular oncotic pressure, which would be aggravated by administration of colloid-free intravenous fluid and would enforce the formation of interstitial edema. Thus, fluids that mainly remain within the vasculature and maintain oncotic pressure are needed to treat acute loss of plasma volume effectively: colloids.

## Intravenous fluids: crystalloids and colloids

### Crystalloids

Crystalloids freely distribute across the vascular barrier. Only one fifth of the intravenously infused amount remains intravascularly [15,16]. Proclaimed by textbooks, a fourfold amount of crystalloid infusion is needed to reach comparable volume effects as achieved with colloid administration. Whereas this is true if the vascular barrier is intact, in patients suffering from capillary leakage ratios from only 1.6:1 to 1:1 (crystalloid to colloid infusion) were observed to reach equivalent effects [29,30]. Nevertheless, colloid treatment resulted in a greater linear increase in cardiac preload and output in septic and nonseptic hypovolemic patients compared with crystalloid administration [31], and its volume expansion lasted longer during acute hemorrhage experimentally [32]. Although currently discussed, regarding the double-barrier concept one could assume that colloids distribute nearly as freely as crystalloids across a seriously impaired vascular barrier. However, volume resuscitation with crystalloid infusions was associated with serious complications, such as respiratory distress syndrome, cerebral edema, and abdominal compartment syndrome in patients with major trauma [33-35] and promotes the development of hyperchloremic acidosis [36]. Even if there is ongoing discussion about the benefits and risks of balanced crystalloid solutions, their use is beneficial to avoid acid-base disorders [25].

### Colloids

The only natural colloid used in clinical matters is albumin. The artificial colloids hydroxyethyl starch (HES) and gelatin are used prevalently in European countries, whereas albumin is applied less commonly [37].

### Albumin

Under physiologic conditions, albumin is the molecule mainly accountable for intravascular osmotic pressure and should be an ideal colloid to restore protein loss from the vasculature. However, as a natural colloid, albumin may cause severe allergic reaction and immunologic complications. Current date concerning albumin use to treat hypovolemia mainly originate from critically ill patients. A Cochrane review of 30 randomized, controlled trials, including 1,419 patients with hypovolemia, showed no evidence for a reduced mortality comparing albumin to crystalloid volume resuscitation. Usage of albumin may contrariwise even increase mortality [38]. More recently, the SAFE Study, including 6,997 patients and comparing albumin to normal saline fluid resuscitation, found neither beneficial effects nor an increased mortality in the albumin group. Additionally, no differences in days of mechanical ventilation or need for renal-replacement therapy were observed [39]. In contrast to isooncotic albumin, which does not influence the outcome of critically ill patients, treatment with hyperoncotic albumin increased mortality [40]. Therefore, administration of isooncotic albumin may be justifiable in particular cases but not as a routine strategy for volume resuscitation.

### Gelatins

Gelatins are polydispersed polypeptides from degraded bovine collagen. The average molecular weight of gelatin solutions is 30,000 to 35,000 Da and their volume-expanding power is comparable. Several studies have examined the pharmacological safety of gelatins. In brief, all preparations are said to be safe in regard to coagulation and organ integrity [15,16] except kidney function. Mahmood et al. demonstrated higher levels of serum urea and creatinine as a more distinct tubular damage in patients treated with 4% gelatin solution compared with hydroxyethyl starch (HES) solutions while undergoing aortic aneurysm surgery [41]. Therefore, use of gelatins is limited in renal-impaired patients.

### Hydroxyethyl starch

Hydroxyethyl starch, an artificial polymer, is derived from amylopectin, which is a highly branched chain of glucose molecules obtained from waxy maize or potatoes. Conservation from degradation and water solubility are achieved by hydroxyethylation of the glucose units. HES solutions are available in several preparations and vary in concentration, molecular weight, molar substitution, C^2^/C^2 ^ratio, solvent, and pharmacologic profile. Although small HES molecules (< 50-60 kD) are eliminated rapidly by glomerular filtration, larger molecules are hydrolyzed to smaller fractions and are partially taken up in the reticuloendothelial system. Although this storage seems not to impair the mononuclear phagocytic system, it is remarkable that low molecular weight HES accumulates less compared with high molecular weight HES [42]. Negative effects of high molecular HES on the coagulation system are well described. Preparations >200 kD lead to a reduction of von Willebrand factor and factor VIII, causing a decreased platelet adhesion. Low molecular weight preparations, such as HES 130/0.4, have only minimal effects on coagulation. HES in balanced solution increases the expression of activated platelet GP IIb/IIIa, indicating an improved hemostasis [43,44]. Focusing on kidney function, an 80% rate of "osmotic nephrosis-like lesions" and impaired renal function were reported in kidney transplant recipients after administration of HES 200/0.62 to brain-dead organ donors [45,46]. In septic patients, usage of 10% HES 200/0.5 correlated with a higher incidence of renal failure compared with crystalloids [47]. Admittedly, HES was administered without regard to exclusion criteria and dose limitations in this study. The most likely pathomechanism of renal impairment by colloids is the induction of urine hyperviscosity by infusing hyperoncotic agents in dehydrated patients. Glomerular filtration of hyperoncotic molecules causes a hyperviscous urine and results in stasis of the tubular flow [48]. Elevated plasma oncotic pressure, regardless of which genesis, is known to cause acute renal failure since more than 20 years [49]. Based on this pathogenesis, all hyperoncotic colloids may induce renal damage, whereas iso-oncotic tetra starch solutions, such as 6% HES 130/0.4, seem not to impair renal function [41,46]. After administration of extremely high application rates (up to 66 liters in 21 days) in patients with severe head injury, no impairment of renal function was observed [50]. In contrast to results of the VISEP study [47], the SOAP study, which included more than 3,000 critically ill septic patients treated with pentastarch and tetrastarch solutions, also showed no higher risk for renal failure [51]. Hydroxyethyl starch was administered in much lower amounts (13 vs. 70 ml/kg) and for a shorter period in the SOAP study. There is evidence that HES also modulates inflammation. Synthetic colloids inhibit neutrophil adhesion to the endothelium and neutrophil infiltration of the lung [52,53].

Furthermore, HES attenuated inflammatory response in septic rats as well as in rats volume resuscitated with HES 130/0.4 during severe hemorrhagic shock by decreasing tumor necrosis factor-alpha, interleukins, and oxidative stress [53,54]. Although advantageous aspects of volume replacement with so-called "modern" isooncotic tetrastarch solutions, in particular in reaching early hemodynamic stability are comprehensible [31,32], data on focussed, adequately powered, prospective clinical trials proving their outcome-relevance are needed.

## Goals and strategies for volume replacement

Because the primary goal of the cardiovascular system is to supply adequate amounts of oxygen to the body and to match its metabolic demands, the target of volume management is to maintain adequate tissue perfusion to ensure tissue oxygenation. Hypovolemia, as well as hypervolemia, decreases tissue perfusion and may result in organ failure [55-59]. Even supplemental oxygen does not improve oxygenation in hypoperfused tissue [60]. Because hypovolemia is a frequent cause for hemodynamic deterioration in critically ill patients, securing an adequate intravascular volume is a cornerstone of hemodynamic management. But how can we assess "adequate" intravascular volume? Because the relation between hemodynamic variables is complex in health already, it is even more complex in disease and their interpretation requires a solid understanding of cardiovascular regulation mechanism.

In hemodynamic unstable patients, basically four functional questions need to be answered. Because the primary goal of resuscitation is to secure tissue oxygenation, the first question is already the most decisive, but also the most difficult one: Is tissue oxygenation adequate? Because representative tissue oxygenation is not measurable directly, primarily three variables are used as surrogates: mixed venous oxygen saturation; central venous oxygenation; and serum lactate. Use, interpretation, and significance of these parameters concerning assessment of tissue oxygenation are discussed elsewhere. In brief, none of them is able to detect tissue oxygen debt definitely, because every single one is influenced by various morbidities and drug interactions [61-64]. The second question is: How can cardiac output (CO), as the main determinate of oxygen delivery, be improved? Or, better representing clinical matters: Is the patient volume responsive? The third question regards the vasomotor tone: Is it increased, decreased, or normal in the hypotensive patient? Fourth, heart work: Is the heart able to sustain an adequate CO when arterial pressure is restored without going into failure [65]?

Usually physicians address these questions by measuring mean arterial pressure (MAP), central venous pressure (CVP), and by observing diuresis [66]. All of these parameters are easy to measure, but actually do not allow to assess hemodynamic instability sufficiently or to differentiate its cause adequately. If disease leads to a decrease of CO, the physiologic reaction of the body, mediated by baroreceptors, is to restore the likewise decreased arterial pressure to maintain cerebral perfusion pressure [67]. This is frequently accompanied by tachycardia, caused by modulation of the sympathetic tone. Hence, hypotension reflects the failure of this compensating mechanism, whereas normotension does not automatically ensure hemodynamic stability [68]. In addition, tachycardia and hypotension can be absent during hypovolaemic shock until intravascular volume loss reaches 20% or more [69,70]. CVP shows a poor correlation to blood volume [71], is inadequate to detect hypovolemia reliably, and most notably cannot sense a decreased cardiac output and tissue oxygen debt in an early state. Furthermore, changes in CVP after volume administration do not allow any conclusions to changes in stroke volume (SV) or cardiac output (CO) [72]. Measuring CVP is therefore inadequate to assess the patient's hemodynamics and to manage volume resuscitation. Because CO is the primary determinate by which oxygen donation to the tissue is varied to match metabolic requirements, the effectiveness of a resuscitation therapy can be evaluated best by continuous monitoring of cardiac output. Several different methods, ranging from the classical indicator dilution techniques to less invasive approaches, such as arterial pulse contour analysis and Doppler techniques, are clinically available. A detailed description and discussion of their individual advantages and disadvantages is beyond the scope of this article and can be found in recent reviews [73,74]. Suitable monitoring techniques for defining treatment strategies are able to assess cardiac output as well as cardiac preload and, first of all, to predict volume responsiveness of the patient, which mostly applies to volumetric and functional parameters utilizing the heart-lung interaction under mechanical ventilation [75-78]. In the past, various studies were published that favored individual concepts of perioperative volume management strategies. Most of them originated from perioperative care and focussed primarily on the treatment in the operating room. Of course, those strategies impact postoperative ICU treatment as well. "Restrictive" strategies were compared with "permissive" or "liberal" ones. However, commonly accepted definitions of "restrictive" or "liberal" fluid strategies do not exist, making those studies nearly incomparable. Investigators normally labelled their traditional standard fluid regimen the "standard" group and compared it with their own restrictive fluid administration model. "Liberal" in one study was already "restrictive" in the other trial and fluid administration followed rigid schemas or different goals. Additionally, endpoints of the given studies varied from postoperative vomiting, pain, or tissue oxygenation to bowel recovery time, which de facto rules out a comparison [79-82]. One of the most cited studies in this regard is the work of Brandstrup et al., who demonstrated that perioperative fluid restriction (2740 vs. 5388 ml) reduced the incidence of anastomotic leakage, pulmonary edema, pneumonia, and wound infection in 141 patients undergoing major colorectal surgery without increasing renal failure rate. Interestingly, a closer look at the infusion protocol reveals a comparison between crystalloid versus colloid fluid administration. The restrictive group received mainly colloids, whereas the liberal group was treated exclusively with crystalloids [79]. All of those studies have in common that no hemodynamic goals were set, which is in contrast to the "goal-directed-therapy (GDT) approach" known most prominently from the study by Rivers et al., in which the authors used central venous pressure, mean arterial pressure, serum lactate, and mixed venous oxygen saturation as goals to optimize the early treatment in septic patients [83]. Further peri- and postoperative studies in surgical patients underline the importance of "functional" hemodynamic goals to improve patients' outcome. In a meta-analysis encasing four prospective randomized trials, cardiac output guided fluid management reduced hospital stay and lessened complication rate [84]. Additionally, interleukin-6 response was attenuated in a colorectal surgery study using a Doppler-optimized goal-directed fluid management [85]. Göpfert et al. reported a reduced time of mechanical ventilation and intensive care unit stay in cardiac surgery patients using the global end-diastolic volume index and cardiac output to manage volume administration [86]. The extravascular lung water index may be a useful tool for GDT, too, and is subject of current discussion [87]. Furthermore, goal-directed fluid therapy reduces inflammation, morbidity, and mortality not only in severe sepsis and septic shock, but also in patients who undergo major surgery [88-90].

## Conclusions

Consolidated findings regarding the endothelial surface layer led to a new comprehension of the vascular barrier. Starlings' principle was adjusted to the "double-barrier concept" and the mechanisms of ESL alteration in critically ill patients seem to play a major role in tissue edema formation. Because glycocalyx diminution leads to an increased capillary permeability, fluid loss toward the interstitial space, commonly considered to be a loss toward the "third space," is one major consequence of ESL degradation. Studies concerning fluid and volume therapy prove an adverse effect of tissue edema formation on organ function and mortality. Therefore, knowledge of the consequences of infusing different types of crystalloids and colloids during physiologic and pathologic states is necessary. Furthermore, fluid and volume administration are two different therapies for two different diagnoses. Dehydration resulting from urine loss, preoperative fasting, and insensible perspiration requires fluid administration primarily based on crystalloid infusions. Intravascular volume deficit, i.e., acute hypovolemia, resulting in a decreased cardiac output requires volume replacement, where colloid administration appears meaningful, although current clinical data are not finally consistent. The right amount of administered volume should be titrated "goal directed" using a strategy based on macro-hemodynamic parameters of flow and volume.

## Competing interests

Daniel A. Reuter is member of the medical advisory board of Pulsion Medical Systems AG and held lectures for B. Braun Melsungen AG and Fresenius Kabi. Alwin E. Goetz is member of the medical advisory board of Pulsion Medical Systems AG, Germany, and held lectures for B. Braun Melsungen AG, Fresenius Kabi, Baxter, and Abbott.

## Authors' contributions

MS, KH, AG and DR enquired the literature and drafted the manuscript. All authors read and approved the final manuscript.
